# Impulse Control Disorders and Other Compulsive Behaviours in Parkinson’s Disease – What Is Current Evidence of Treatment?

**DOI:** 10.1192/bjo.2025.10794

**Published:** 2025-06-20

**Authors:** Agnieszka Gross

**Affiliations:** Lancashire and South Cumbria NHS Trust, Blackburn, United Kingdom

## Abstract

**Aims:** Parkinson’s disease (PD) is a progressive neurodegenerative disorder that primarily affects motor function but also impairs cognitive and emotional regulation. Dopaminergic therapy, commonly used to treat motor symptoms, can lead to complications such as impulse control disorders (ICDs), compulsive behaviours, and dopamine dysregulation syndrome (DDS). These complications often result in significant deterioration of patients’ quality of life. This dissertation aims to investigate effective interventions for managing ICDs and compulsive behaviours in PD patients on dopaminergic treatment while minimizing the risks of motor symptom deterioration and other neuropsychiatric consequences.

**Methods:** This systemic literature review examines a wide range of interventions for managing ICDs and compulsive behaviours in PD patients. A comprehensive search was conducted across major medical and psychological databases to identify studies evaluating pharmacological and non-pharmacological treatments. The review focused on interventions such as clonidine, atomoxetine, cognitive behavioural therapy (CBT), and subthalamic nucleus deep brain stimulation (DBS). Inclusion criteria were studies published in peer-reviewed journals within the last two decades that specifically addressed the treatment of ICDs and compulsive behaviours in PD patients receiving dopaminergic therapy.

**Results:** The review found that several interventions show promise in managing ICDs and compulsive behaviours without exacerbating motor symptoms. Clonidine and atomoxetine, both of which affect norepinephrine pathways, have been identified as potentially effective pharmacological options for controlling impulsivity and compulsive behaviours. CBT has been highlighted as an effective psychological intervention, particularly in improving patients’ coping mechanisms and reducing maladaptive behaviours. Additionally, deep brain stimulation of the subthalamic nucleus has demonstrated positive effects on reducing impulsivity in PD patients, though its use remains primarily for motor symptom management. However, the evidence remains inconclusive regarding the optimal approach for balancing treatment of both motor and non-motor symptoms.

**Conclusion:** The management of ICDs and compulsive behaviours in PD patients remains complex due to the delicate balance between controlling motor symptoms and minimizing dopaminergic side effects. While pharmacological interventions such as clonidine and atomoxetine, as well as non-pharmacological treatments like CBT and DBS, offer potential benefits, further research is needed to refine these approaches. Additionally, more tools are required for the comprehensive risk assessment of ICDs and compulsive behaviours to guide clinicians in tailoring treatments that safeguard both motor function and mental well-being, ultimately improving patient quality of life.

